# Estimation of Grazing Activity of Dairy Goats Using Accelerometers and Global Positioning System

**DOI:** 10.3390/s22155629

**Published:** 2022-07-27

**Authors:** Youssef Chebli, Samira El Otmani, Jean-Luc Hornick, Jérôme Bindelle, Jean-François Cabaraux, Mouad Chentouf

**Affiliations:** 1 Regional Center of Agricultural Research of Tangier, National Institute of Agricultural Research, Avenue Ennasr, BP 415 Rabat Principale, Rabat 10090, Morocco; samira.elotmani@inra.ma (S.E.O.); mouad.chentouf@inra.ma (M.C.); 2 Department of Veterinary Management of Animal Resources, FARAH, Faculty of Veterinary Medicine, University of Liège, 4000 Liège, Belgium; jlhornick@uliege.be (J.-L.H.); jfcabaraux@uliege.be (J.-F.C.); 3 Precision Livestock and Nutrition Unit, TERRA Teaching and Research Center, Gembloux Agro-Bio Tech, University of Liège, 5030 Gembloux, Belgium; jerome.bindelle@uliege.be

**Keywords:** goat, sensor, GPS collar, grazing activity, intake, Mediterranean woodland

## Abstract

The recent development of advanced electronic sensors to monitor and record animal grazing activity provides a real opportunity to facilitate understanding of their behavioral responses. This study aimed to characterize the grazing activity and protein–energy requirements of grazing dairy goats in a Mediterranean woodland in northern Morocco by combining two sensors, the Global Positioning System (GPS) and three axis accelerometers. An experiment was conducted in a representative woodland with eight dairy goats. Measurements were undertaken during the three main grazing seasons (spring, summer, and autumn) for two consecutive and contrasting years. Grazing activity parameters were assessed using GPS collars and leg position sensors. The results showed that grazing time was higher in spring (57% and 59%) than in summer (39% and 36%) and autumn (41% and 45%), respectively, for the dry and wet years (*p* < 0.001). During the two studied years, the daily horizontal distance traveled by the goats increased from spring (about 4.5 km) to summer (about 6.5 km) and autumn (about 7.4 km), while greater daily vertical distances were recorded over similar distances during summer–autumn. Several protein–energy intakes that were insufficient to cover the requirements of the grazing goats were recorded, especially in summer. The combination of GPS collars and accelerometers contributed to a better understanding of the grazing activities of dairy goats in the studied Mediterranean woodland. These findings provide useful data on the protein–energy balances of dairy goats and offer additional information that could be useful for herders and managers to enhance goat-feeding strategies and guarantee high-performance in the semi-extensive traditional goat farming system.

## 1. Introduction

In the Mediterranean region, browsing on natural pastures is the main source of feed for domestic goats [1,2,3]. Livestock farming, particularly grazing goats, constitutes the prevalent agricultural activity in the mountainous areas of the southern Mediterranean Basin. Due to their high adaptability to harsh environments, goats do not need to receive any significant investments [4,5,6]. In addition, they contribute to the livelihoods of poor herders and provide several benefits to mountainous populations [3,7]. Currently, the southern Mediterranean region of northern Morocco is suffering from severe vegetation degradation, which is mainly due to overgrazing, deforestation, and land reconversion [8]. Such degradation of biodiversity could affect grazing patterns, ruminant feeding, and animal performance, especially that of goats.

Previous studies have listed plant species ingested by goats in Mediterranean woodlands, [3,9,10], and some studies focused on their nutritional values [2,6,11]. As reported by several authors [12,13,14], forage availability and quality are characterized by seasonal variations that can affect plant selection and production performance in grazing goats. There are, however, differences in the degrees of these variations depending on each region’s climate, vegetation, and animal management factors.

Grazing goats in southern Mediterranean woodlands are currently affected by climate change effects (long dry period and reduced rainfall), resulting in reduced goat performance and productivity [8]. To address this issue, some farmers have adopted a semi-extensive system to meet animals’ nutritional requirements and to ensure the productivity of their dairy goat production systems.

Most of the studies focusing on the grazing activity of animals in the Mediterranean region are based on conventional approaches, such as direct observation [2,6,15]. Relying exclusively on direct behavioral observations is impractical and leads to incomplete evaluations of animal behavior [16]. Continuous observation of grazing activities is inapplicable in situations where animals cannot be directly monitored. Furthermore, this method is labor-intensive and time-consuming [17]. The use of recently developed advanced electronic sensors, such as the Global Positioning Systems (GPS) and axis accelerometers (e.g., leg sensors), to monitor and record behavior across different spatiotemporal scales provides a real opportunity to facilitate understanding of animal behavioral responses. Monitoring grazing activity by using sensors can gather more accurate and reliable information, which could be used to derive management decisions to optimize animal performances.

Grazing animals are highly dependent on forage availability, which is driven by climate. It is expected that droughts and floods will become more frequent in Mediterranean countries due to climate change [18,19]. Drought is still a major hindrance for sustainable development. During the last three decades, northern Morocco has seen more than four drought periods, which increased the water deficit and led to serious problems in rural areas, such as natural resource degradation and increases in the poverty of local communities [8]. Precipitation is one of the main factors influencing grazing strategies, which manifest through animal mobility and grazing behavior in various ways [20]. Such strategies may be illustrated by longer distances traveled and longer durations spent in search of palatable species, which can increase the energy expended by grazing animals. The extra energy expenditure related to the physical activity of animals can affect their production performance and may be influenced by seasonal variations in grazing activities [21]. Analyses of long-term (seasonal and annual) grazing activities could provide important insights into goats’ responses to both environmental and climate variations.

There is currently little information available on the seasonal and inter-annual variations in the grazing activities of goats. In addition, the seasonal changes in protein–energy balances in accordance with the nutrient supply and dietary requirements of goats in grazing situations are not well understood. These protein–energy balances could be used as the basis for supplementation strategies.

The purpose of this study was therefore to investigate the temporal variations in the grazing activities of dairy goats using accelerometers (leg sensors) and GPS. Moreover, the temporal variations in the browsed diet quality and protein–energy balances were calculated based on the available data regarding the feeding behavior of goats and forage quality combined with data gathered from sensors. This was undertaken through a case study of dairy goats browsing in a mountainous woodland in the southern Mediterranean region of northern Morocco.

## 2. Materials and Methods

### 2.1. Study Woodland

**Figure 1:**
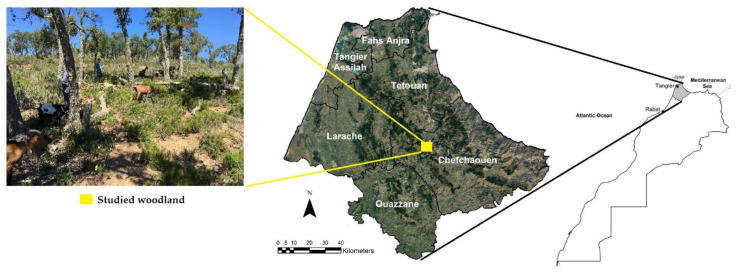
Map of the study area and photo of the studied woodland (northern Morocco).

The study was conducted in an oak woodland in Chefchaouen province (35°08′ N; 5°18′ W; 300 to 520 m a.s.l), located in northern Morocco. The climate in this region is influenced by the Mediterranean Sea and dominated by humid to sub-humid conditions that manifest as dry summers and wet winters. The woodland was studied during two consecutive years that contrasted in terms of precipitation (270 mm in 2016 (dry year) and 755 mm in 2017 (wet year)) [8] (Table 1). The study area was in a mountainous region mainly covered with shrub species and oak trees (Figure 1). The woodland includes *Quercus suber* L., associated with shrubs dominated by *Arbutus unedo* L., *Cistus* species (including *C. crispus, C. monspeliensis* and *C. salviifolius*), *Lavandula stoechas* L. and *Pistacia lentiscus* L.

### 2.2. Experimental Animals and Design

The main dairy goat breed (*Capra aegagrus hircus*) in the study area is alpine. Eight alpine dairy goats (42 ± 2.5 kg live weight (LW) and an average age of 36 ± 6 months) were selected from typical dairy farms breeding from goat flocks grazing year-round in the studied woodland, except in the winter. The flock was chosen from among the most representative in the studied woodland. The goat herds are mainly herded by the farmers themselves or by a family member (a young boy or girl). Goat herders guide their flocks throughout grazing itineraries each day. After arriving in the woodland, goats have free access to the rangelands and their movements are independent of the herders’ decisions. During winter, grazing in the forest pasture is restricted; goats only graze around the farm and in fallow land, which justified the exclusion of this season from the study. After grazing, goats are returned to their shed, where they are individually tethered to limit their fighting for served supplementation. Every day, each goat was equally provided with barley and faba beans during spring, summer and autumn of both years (0.6, 0.3 and 0.7 kg DM/head a day, respectively). For each experimental goat, the individual intake of concentrate was measured daily by weighing the corresponding orts after each meal. Goats were manually milked indoors in the goat shed daily at 6:00 am. The milk production of the experimental goats was recorded daily during spring and summer, which correspond to the milking periods. The experiment was conducted during three consecutive days in each of the three grazing seasons (spring, summer and autumn) of the dry (2016) and wet (2017) years. A total of 72 goats were measured annually during the three grazing seasons.

### 2.3. Collection of Feeding Behavior Data

Direct observation was used as a method to evaluate the composition of the browsed diets of the goats [10,23]. Before starting direct observation, a three-day familiarization period was necessary to accustom the flocks to the presence of observers, as detailed by Bonnet et al. [24] and Meuret and Provenza [25].

Measurements concerned the number of bites and the botanical composition of each part of the plant consumed by goats. Data were collected by observing each goat for 10 min, three times a day. To determine the average bite mass (BM), 100 hand-plucked samples per plant species, similar to those consumed by goats, were imitated seasonally [26]. The hand-plucked samples were collected in special bags and dried in an oven at 40 °C to constant weight to obtain the average dry matter of each BM per consumed plant species. The grazed diet composition was calculated as the percentage of each consumed species using the number of bites per plant and the average BM of each plant species. The number of bites and the time spent grazing were used to calculate the bite rate (BR, number of bites/min). The intake rate (IR, g DM/min) on the pasture was estimated as the product of the BR and BM [27]. The daily dry matter intake (DMI, g/kg LW^0.75^) on the rangeland was calculated as the product of the average IR in accordance with the daily grazing (eating) time, considering the seasonal variations in LW.

### 2.4. Behavioral Measurements

**Figure 2:**
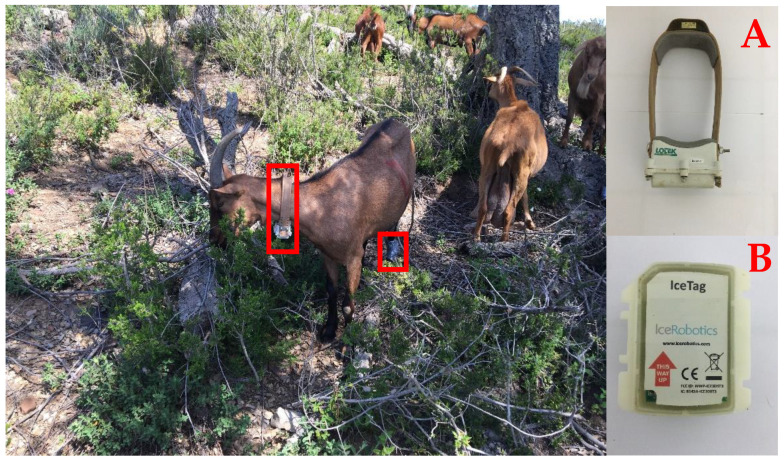
Experimental dairy goat fitted with a GPS collar on the neck (**A**) and with a sensor placed on the rear left leg (**B**) in the studied Mediterranean woodland in northern Morocco.

To estimate the locomotion activities (speed and travelling distances (horizontal and vertical)), a 3300SL GPS collar (Lotek Wireless, Newmarket, ON, Canada) was placed on the neck of each experimental goat. Collars were programmed to acquire a GPS fix every 5 min (Figure 2). GPS collar data were analyzed using GPS software (Model 3000 Host, Lotek Wireless, Newmarket, ON, Canada). The obtained coordinate system (UTM WGS84) was converted to the Moroccan Transverse Mercator using ArcMap 10.x (ESRI, Redlands, CA, USA). The data management tools in ArcMap were used to calculate the coordinates (x, y) in meters for each fixed record. The horizontal (D_H_) and vertical (D_V_) distances traveled were also estimated. D_H_ was calculated using Euclidean geometry between two consecutive pairs of fixed locations A (x_1_, y_1_) and B (x_2_, y_2_), with $D_{H}=\sqrt{{\left(x_{2}-x_{1}\right)}^{2}+{\left(y_{2}-y_{1}\right)}^{2}}$. D_V_ was calculated from the difference in altitude between successive pairs of altitudinal positions (z_1_, z_2_). The speed of goats during grazing was calculated by dividing the total traveled distance D_T_ $\left(D_{T}=\sqrt{{\left(D_{H}\right)}^{2}+{\left(D_{V}\right)}^{2}}\right)$ by the time interval that elapsed between two consecutive positions.

To estimate the number of steps and the time spent lying and standing, a leg sensor (IceTag, IceRobotics Ltd., Scotland, UK) was placed on the rear left leg of each experimental goat (Figure 2). IceTag data were analyzed using IceManager software (IceRobotics Ltd., Scotland, UK). Standing results from the IceTags (tri-axial accelerometers) included both grazing and non-grazing times. The lying position (when the sensor was horizontal) was a unique activity identified without grazing. The calibration and classification tree analyses were performed to predict the grazing activities of each experimental dairy goat (grazing, lying, resting while standing and walking) [28,29]. Therefore, IceTag data were averaged to the same intervals as the GPS collars. 

A trial was conducted in order to use the data from the GPS collars and leg sensors to estimate times spent grazing/eating, as well as other grazing activities. The calibration study involved the visual observation of eight goats equipped with GPS collars and IceTags over a 3 day period. The observed grazing activities were timed manually, as precisely as possible, to serve in the validation of GPS collar and leg sensor data and the model construction [30].

Classification and regression tree (CART) analysis was employed to predict the behavior of the experimental goats using the motion index (a proprietary metric for the overall leg activity as measured in three dimensions), the time spent standing and lying, the number of steps acquired from the leg sensors and fixed location activities (x-activity, y-activity), the horizontal distance traveled and the time spent with heads down detected from the GPS collars. These variables were used as predictors and the grazing activity was considered as the target variable. In each season, time spent in various grazing or non-grazing activities (e.g., lying, standing and walking) was expressed as proportions (%) of the total time spent across all activities.

### 2.5. Browsed Diet Quality

The temporal variations in the browsed diet quality were determined based on the available data for the chemical composition and digestibility of the forage ingested [11] and the diet composition of the experimental goats [3]. These studies were conducted in the same studied woodland during the same periods.

For the forage quality, the analyses were performed on three independent samples of the hand-plucked forage of each plant species consumed by the experimental goats during each grazing season across the two consecutive years. The investigated parameters for the diet quality were the dry matter (DM), organic matter (OM), crude protein (CP), neutral detergent fiber (NDF), acid detergent fiber (ADF), acid detergent lignin (ADL), condensed tannins (CT) and organic matter digestibility (OMD). The metabolizable energy (ME; MJ/kg DM) of each consumed plant species was calculated based on the dry matter digestibility (DMD). Forage quality analyses and digestibility are described fully by Chebli et al. [11].

### 2.6. Protein–Energy Balance Measurements

The daily metabolizable energy requirement for the maintenance (MEm) of the goats was considered to be 424.2 kJ/kg LW^0.75^ [31]. The energy cost for locomotion is relatively well-defined for domestic goats [32]. To estimate this energy, the equations proposed by Lachica and Aguilera [33] were used, as follows: 3.35 and 31.7 J/kg LW per meter for the net energy cost of horizontal and vertical locomotion, respectively. The physiological conditions (pregnancy and lactation periods) of the goats were also considered in the energy balance calculations. For pregnancy, an additional 318 kJ/kg LW^0.75^ for the ME in the last two months of gestation is recommended to be taken into account [31]. Consequently, the ME required for pregnancy was added during the autumn. The additional energy requirement for milk production was estimated using another formula, as follows: 1.23 Mcal per kg of produced milk for 3.5% of fat in milk [31].

The protein balance calculation concerned the daily crude protein (CP) requirements for each goat according to its physical and physiological conditions. In the current case, the NRC [31] suggest about 108 g CP to cover the daily maintenance requirements of grazing goats in mountainous pastures (high physical activity) and additional protein requirements of 68 g CP/kg of milk produced during lactation periods and 82 g CP/goat during pregnancy.

### 2.7. Data Analysis

Data were submitted to analysis using SAS software (SAS version 9.x, New York, NY, USA). Before analysis, data expressed in percentages were arcsine-square root-transformed to normalize the distribution [34]. Browsed-diet-quality data were analyzed according to the GLM procedure. The daily DMI and grazing activities were evaluated according to the PROC MIXED procedure. Individual goats were considered as random factors to prevent this variance from being incorporated into the error term of the analysis. The models contained two fixed variables: the seasons (i.e., spring, summer and autumn) and year (i.e., dry (2016) and wet (2017)) and their interaction. Pair-wise comparisons were performed using the Tukey test. All tests were considered statistically significant at *p* < 0.05.

## 3. Results

### 3.1. Diet Quality and Dry Matter Intake from Pasture

The daily DMI and the chemical composition, in vitro organic matter digestibility (IVOMD) and ME of the browsed diet of the grazing dairy goats in the woodland are summarized in Table 2. Overall, the diet NDF content in the dry and wet years was lower in spring (401 and 381 g/kg DM, respectively) than autumn (456 and 462 g/kg DM, respectively) and summer (483 and 488 g/kg DM, respectively) (*p* < 0.001). The diet ADF concentration was higher for the summer of 2016 (318 g/kg DM; *p* < 0.001), and it was similar for the summer and autumn of 2017 (about 324 g/kg DM), while it was at its lowest level in the spring of 2016 and 2017 (261 and 245 g/kg DM, respectively; *p* < 0.001). Likewise, the acid detergent lignin (ADL) in the selected diet was at its lowest level in spring of 2016 and 2017 (149 and 133 g/kg DM, respectively). No significant differences in the diet ADF content were found between years (*p* = 0.494). In the dry year, the diet CP content was greater in spring (107 g/kg DM; *p <* 0.001) compared to summer (81.9 g/kg DM) and autumn (89.6 g/kg DM), while the CP content was similarly higher in spring and summer (about 96.5 g/kg DM; *p <* 0.01) compared to autumn (94.8 g/kg DM) during the wet year. The CT of the selected diet increased from spring to summer and autumn in both studied years (*p <* 0.001), while the diet IVOMD content significantly decreased from spring to summer and then slightly increased in autumn (*p <* 0.001).

The ME of the selected diet was higher in spring in both years (about 9 MJ/kg DM; *p <* 0.001) compared to summer and autumn, which were similar (about 6.5 MJ/kg DM).

Across dry and wet years, the daily DMI was greater in the spring (69.2 and 73.0 g/kg LW^0.75^, respectively; *p <* 0.001), while it was similarly lower in summer and autumn (values ranging from 45 to 60 g/kg LW^0.75^).

### 3.2. Grazing Activities

The behaviorial activities of the goats under grazing conditions are summarized in Table 3. All studied parameters were influenced by season, year and their interactions (*p* < 0.05). The percentage of time spent lying was more important in summer in the dry and wet years (13% and 15%, respectively) in comparison to autumn (11% and 9%, respectively) and spring (4% and 5%, respectively). Consequently, more time spent standing (including grazing and walking activities) was recorded in spring. The number of steps was similarly higher during summer and autumn compared to spring in 2016, while it increased from spring to summer and autumn in 2017. Daily horizontal distances traveled by the grazing goats during the dry and wet years increased from spring (4.36 and 4.62 km, respectively) to summer (6.89 and 6.15 km, respectively) and autumn (7.76 and 7.06 km, respectively), while greater daily vertical distances were recorded over similar distances during summer–autumn in 2016 and 2017 (about 0.600 and 0.550 km, respectively). The highest speeds were recorded during spring in 2016 and 2017 (0.198 and 0.213 m/s, respectively). In both years, the duration of the grazing day was higher in summer and autumn in 2016 (about 10.8 h) and in summer in 2017 (11.5 h), while the lowest values were recorded in spring in 2016 and 2017 (8.19 and 7.25 h, respectively). The proportion of time spent grazing (grazing time) was higher in spring in the dry (57%) and wet (59%) years compared to summer (39% and 36%, respectively) and autumn (41% and 45%, respectively). Goats rested in the standing position for longer times on average in spring in the dry year (23%) and in summer in the wet year (30%). Irrespective of the year, goats spent more time walking in autumn than in spring and summer.

### 3.3. Protein–Energy Requirements and Balances

**Figure 3:**
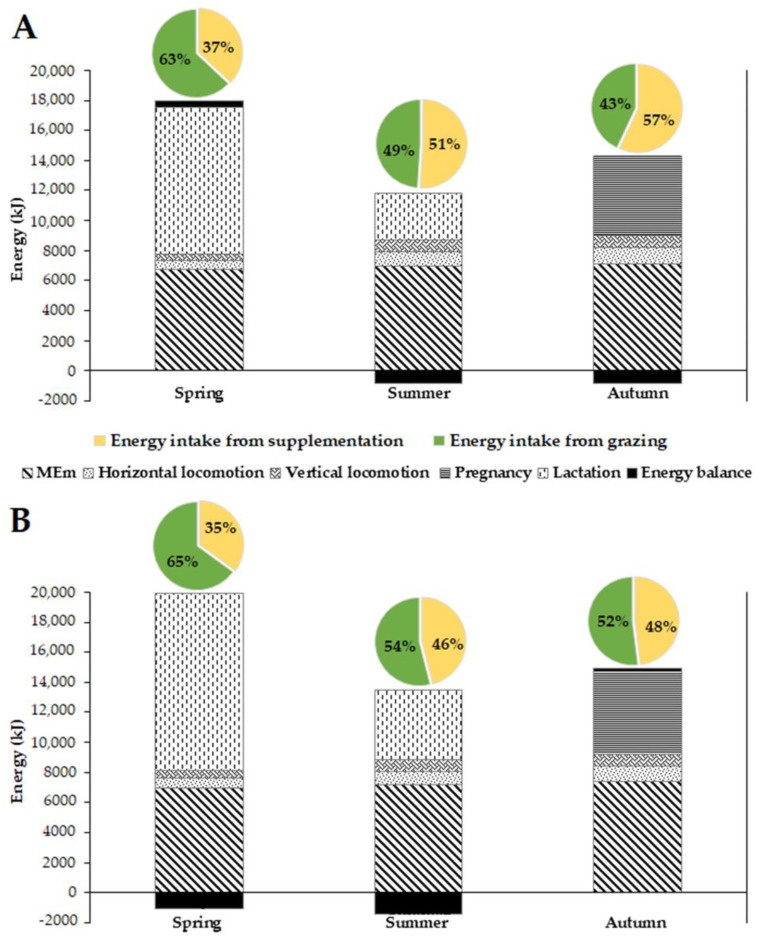
Estimation of temporal variations in energy requirements (maintenance, locomotion, pregnancy and lactation) and energy balance for dairy goats browsing in a Mediterranean woodland in northern Morocco. (**A**), dry year; (**B**) wet year. MEm, daily metabolizable energy requirement for maintenance. Pie charts represent the intake contributions from grazing and supplementation.

Figure 3 shows the seasonal energy requirements and energy balance for dairy goats under grazing conditions, considering forage and supplementation intake. Across seasons in both years, MEm and the horizontal locomotion energy requirement increased, while lactation energy requirements decreased from spring to autumn. The MEm was higher in autumn and lower in spring in both years. The milk yield of the alpine goats was measured during the milking period. The energy cost of lactation was added during the spring and summer because these seasons corresponded to the lactation periods in the study area. The average daily milk production of the dairy goats was measured as 1.9 and 0.6 kg/goat during spring and summer in the dry year, respectively, and 2.3 and 0.9 kg/goat during spring and summer in the wet year, respectively. Consequently, a higher lactation energy requirement for milk production was recorded in spring in 2016 and 2017 (9785 and 11,844 kJ/goat a day, respectively). In the summer, the additional requirement energy for daily milk production was estimated at 3090 and 4635 kJ/goat in 2016 and 2017, respectively. The daily locomotion energy (horizontal and vertical) was higher in summer and autumn (>1650 kJ/goat) and lower during spring (<1150 kJ/goat) in both years. The vertical locomotion energy increased from spring to summer and then slightly decreased in autumn. An inverse tendency was observed for the energy intake from grazing. The herder provided supplementation to the goats with the same quantity of grains during the same season in each year; consequently, the energy intake from supplementation was the same throughout the year. The highest daily supplementation energy was recorded during autumn (7761 kJ/goat), followed by spring (6653 kJ/goat) and summer (5544 kJ/goat). Pasturing contributed more to the energy intake of the goats during spring (63 to 65%) compared to summer (49 to 54%) and autumn (43 to 48%) in the dry and wet years, respectively.

**Figure 4:**
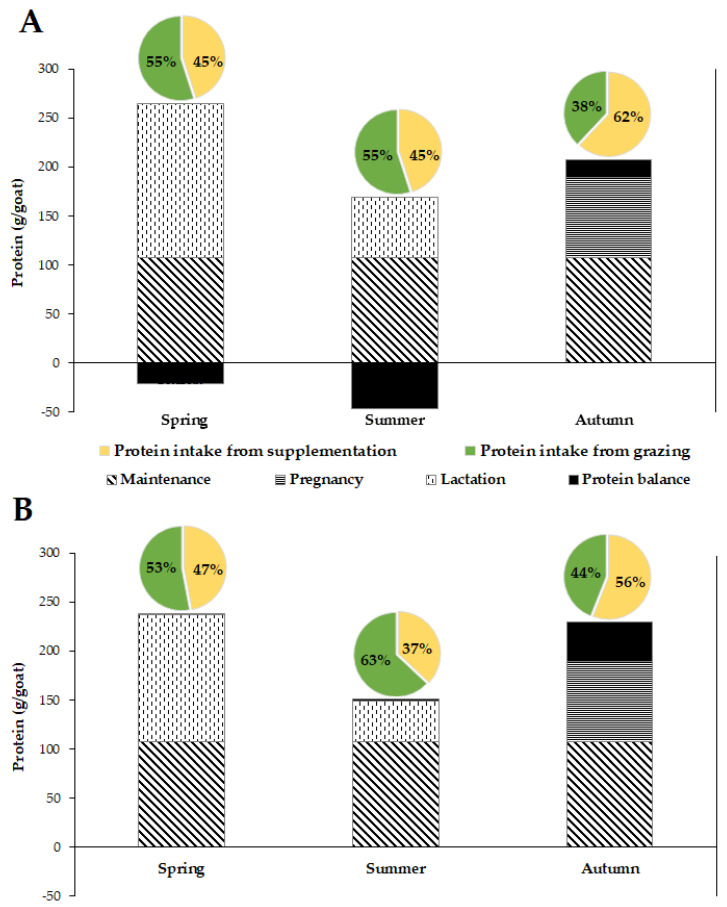
Estimation of temporal variations in protein requirements (maintenance (for goats with high activity), pregnancy and lactation) and protein balance for dairy goats browsing in a Mediterranean woodland in northern Morocco. (**A**), dry year; (**B**) wet year. Pie charts represent the intake contributions from grazing and supplementation.

The protein requirements for goats in the current case study are detailed in Figure 4. During the dry year, the protein balance was in deficit, but it increased from spring to summer and it was in surplus during autumn. However, during the wet year, the protein balance was in excess across the seasons. Protein intakes from grazing were approximately similar in spring in both years (55%). During the summer and autumn of the wet year, the contribution of grazing to the total protein intake was higher compared to the dry year, but it decreased by 17% from summer to autumn.

## 4. Discussion

### 4.1. The Browsed Diet Quality and Dry Matter Intake

The nutritional value of the browsed diet showed wide variability in macronutrient contents throughout the seasons and years. The seasonal and inter-annual variations in the quality of the goats’ grazed diet were also confirmed, with better quality during spring than summer and autumn [11]. The diet NDF and ADF values increased significantly with advances in the maturity of the consumed plant species [35]. The diet quality in spring was characterized by low ADL and CT contents but greater CP, IVOMD and ME values compared to those observed in summer and autumn. The values were generally higher during the wet year compared to the dry year. The IVOMD and ME concentrations in the plant species were inversely related to the cell wall compounds and positively related to the CP and OM ratios [6,11]. Therefore, plant species had low IVOMD and ME values, and both were generally associated with high NDF, ADF, ADL and CT values [11].

The daily DMI results were within the ranges advised by Luo et al. [36] and Dove [37] for lactating goats (31.7–151.3 g/kg LW^0.75^). Spring seasons were characterized by high availability of most palatable species, which could explain the higher DMI (approximately 70 g/kg LW^0.75^) and the greater contribution of grazing to the total energy intake (approximately 65%) compared to summer and autumn (less than 55%). In addition to forage availability, the higher DMI on the pasture could also be explained by the grazing behavior of the goats in each feeding station and the herder strategy during grazing [25].

Based on NRC [31] references, the DMI from grazing provided sufficient energy to cover the MEm requirements of goats in spring in 2016 and 2017 (167% and 175%, respectively), but it was insufficient during summer (77% and 90%, respectively) and autumn (81% and 97%, respectively).

### 4.2. Grazing Activities

The results of this study correlated with the seasonal and interannual variations of goat grazing behavior in forest pastures [3,15]. As stated in several studies, during the seasons with low forage availability, goats browse for longer durations in the woodland to increase their DMI [3,14]. Grazing behavior data indicated that, with decreased forage availability and increased temperature, the lying time, number of steps, locomotion activities (horizontal and vertical distance) and walking time increased; and, conversely, the standing time (including grazing and resting times), speed and grazing time decreased. During seasons with low forage availability, goats spent more time moving between feeding stations to maximize their instantaneous intake rate. Therefore, based on these results, it seems that the reductions in the forage availability and nutritional value of plant species during summer and autumn could explain the greater distances traveled and grazing day durations. Charnov [38] reported that reduced forage availability led to reduced time spent by animals at each feeding station and, consequently, increased locomotion distance and, hence, numbers of steps. As observed during the summer and autumn, goats tended to balance lower forage intake rates and longer lying and walking times by extending the daily grazing duration. This extended browsing time mainly depended on herder decisions and weather conditions [3,15]. There is strong evidence that seasonal variations in goats’ itinerary length are related to changes and shifts in forage quality and availability [39].

In the studied pasture, higher temperatures were recorded in summer and autumn than in spring (on average, 28 °C in summer and 24 °C in autumn vs 18 °C in spring). During these seasons with higher temperatures, goats rested while lying down longer (9 to 13% of their time), mainly during the middle of the day in the shade of oaks. This is in accordance with the studies by Tovar-Luna et al. [30] and Askar et al. [40], who confirmed that the longest lying times were observed when the temperature increased. It also agrees with Ferreira et al. [41] and Tolu et al. [42], who found that ruminants are more active in the morning and during the afternoon, whereas in the middle of the day they prefer resting in a lying position.

The spring season was characterized by good weather conditions [3], high forage availability and high nutritional values in plant species [11], which made goats more active (increase in their speed) and resulted in low moving distances between feeding stations (decrease in locomotion distance).

The higher temperature recorded during the middle of the day in the summer of the wet year compared to the dry year could explain the longer lying and standing times (without grazing) and the extended duration of the grazing day, as well as, conversely, the decreased grazing time, locomotion distance, speed and walking time.

### 4.3. Protein–Energy Requirements and Balances

The results showed that, between the two years, the energy balance of the grazing goats (including supplementation) was slightly in excess (<3%) of the estimated energy requirements in the spring of 2016 and autumn of 2017 and, conversely, in deficit in the other seasons (5 to 11%). The deficit recorded during the spring and summer of the wet year could be explained by the increase in daily milk production (an increase of 400 and 300 g/goat a day, respectively) compared to spring and summer of the dry year, which entailed increased requirements for lactation energy (2060 and 1545 KJ/goat a day, respectively). In addition, this deficit could be also explained by the higher LW recorded in the wet year (which was higher by 1.5 to 2.3 kg LW) compared to the dry one. The diminutive excess of energy observed in spring of 2016 could be explained by the drop in milk production, reducing the energy required for lactation in this season. Despite the high supplementation during autumn (700 g/goat a day), the low DMI on rangeland recorded during the autumn of 2016 could explain the slight deficit observed (approximately 1.3%). Furthermore, the longer distances traveled by goats during summer and autumn increased their energy requirements for locomotion more than in spring.

Grazing with supplementation provided sufficient protein to cover maintenance, lactation and pregnancy requirements for goats during the wet year. In contrast, during the dry year, the protein balance was in deficit in spring and summer (8% and 27%, respectively), while it was in surplus in autumn (9%). The positive protein balance noted during the wet year could be explained by the increase in DMI and the high CP concentration in the selected plant species. The reduced DMI and the high lactation protein requirements seem to explain the negative protein balance recorded during spring and summer of the dry year, while the positive balance recorded during autumn was due to the absence of protein requirements for milk production and the availability of sufficient protein for pregnancy.

In general, these results are consistent with previous research that reported insufficient energy and protein intakes from forest pastures to cover the requirements of grazing goats during dry seasons [43,44,45].

In summary, increases in the duration of the grazing day should be arranged to increase goats’ DMI with the increase in their diet supplementation during all seasons in accordance with their energy requirement in order to enhance the performance of grazing goats.

## 5. Conclusions

The results of the current study showed that the grazing season affected the browsed diet quality and the grazing contribution to the total energy and protein intake. The primary factor affecting the grazing time was the quantity of forage consumed by goats during each browsing period, which depended on the forage availability in the studied woodland. During seasons with low forage availability (summer and autumn), grazing behavior data indicated that lying time, numbers of steps, locomotion activities and walking time increased and, conversely, standing time, speed and browsing time decreased.

The present study demonstrated the complex dynamics of the grazing activity of goats browsing on heterogeneous southern Mediterranean woodland. Northern Moroccan woodland provides an essential and free source of energy and protein for grazing dairy goats.

The combination of accelerometers and GPS provided the opportunity to monitor and understand the grazing activities of goats in a mountainous woodland in northern Morocco. With regard to the utility of such findings, it is necessary for the government to play a prominent role in the promotion and inclusion of new, high-tech applications in traditional pastoral practices, deploying low-cost precision tools to accurately monitor livestock grazing behavior and provide adaptive management systems, where decisions can be integrated with monitoring at various spatial and temporal scales. In addition, differences in seasonal and inter-annual grazing activities may provide opportunities and tools for herders and managers to enhance the feeding strategies of dairy grazing goats and, hence, guarantee the sustainability of forest pasture. The detailed estimation of the protein–energy balance highlighted the need to reach an appropriate balance between intake and requirements under grazing conditions, whether by improving grazing conditions or diet supplementation; this will obviously depend on farmers’ budgets. Poor farmers (small herders) could use crop residues and supplementation with alternative feed resources, such as olive cake and cactus cladodes, widely available and free in the region, to diversify dairy goats’ diets and satisfy their nutrients needs.

## Figures and Tables

**Table 1:** Seasonal variations in average precipitation (mm) and air temperature (°C) for dry (2016) and wet (2017) years in the studied area (northern Morocco) [22].

Season	Month	2016 (Dry Year)		2017 (Wet Year)	
		Precipitation	Temperature	Precipitation	Temperature
**Winter**	December	0.00	15.81	140.00	13.56
	January	40.00	16.74	39.00	13.97
	February	21.00	15.62	94.00	15.64
**Spring**	March	20.50	17.26	39.00	17.98
	April	45.00	20.90	87.00	20.70
	May	58.30	19.63	25.00	20.47
**Summer**	June	3.40	23.95	7.40	24.90
	July	12.20	31.58	1.50	30.95
	August	5.20	31.06	14.00	30.31
**Autumn**	September	0.00	28.88	0.00	23.27
	October	48.00	24.79	95.00	21.42
	November	15.00	18.30	214.00	17.68

**Table 2:** Temporal variations in the chemical composition (g/kg DM), ME (MJ/kg DM) and IVOMD (g/kg) of the browsed diet, along with the daily DMI on pasture (g/kg LW^0.75^), of experimental dairy goats browsing in a Mediterranean woodland in northern Morocco (data based on Chebli et al. [3,11]).

	Dry Year (2016)					Wet Year (2017)						*p*-Value		
	Spring	Summer	Autumn	SEM	*p*-Value	Spring	Summer	Autumn	SEM	*p*-Value	SEM	S	Y	S × Y
**OM**	920 ^b^	951 ^a^	950 ^a^	0.994	<0.001	935 ^b^	958 ^a^	961 ^a^	0.825	<0.001	0.696	<0.001	<0.001	<0.001
**NDF**	401 ^c^	483 ^a^	456 ^b^	2.38	<0.001	381 ^c^	488 ^a^	462 ^b^	3.21	<0.001	2.00	<0.001	0.009	<0.001
**ADF**	261 ^c^	318 ^a^	312 ^b^	1.85	<0.001	245 ^b^	324 ^a^	323 ^a^	2.62	<0.001	1.60	<0.001	0.494	<0.001
**ADL**	149 ^b^	155 ^a^	153 ^a^	0.465	<0.001	133 ^c^	165 ^b^	178 ^a^	1.40	<0.001	0.753	<0.001	<0.001	<0.001
**CP**	107 ^a^	81.9 ^c^	89.6 ^b^	0.764	<0.001	96.3 ^a^	96.7 ^a^	94.8 ^b^	0.263	0.009	0.410	<0.001	<0.001	<0.001
**CT**	31.6 ^c^	94.5 ^b^	101 ^a^	2.19	<0.001	24.6 ^c^	89.2 ^b^	99.6 ^a^	2.32	<0.001	1.60	<0.001	<0.001	0.007
**ME**	9.09 ^a^	6.58 ^b^	6.55 ^b^	0.082	<0.001	9.35 ^a^	6.51 ^b^	6.70 ^b^	0.089	<0.001	0.060	<0.001	<0.001	<0.001
**IVOMD**	630 ^a^	450 ^c^	460 ^b^	5.67	<0.001	643 ^a^	462 ^c^	473 ^b^	5.70	<0.001	4.03	<0.001	<0.001	0.300
**DMI ^1^**	69.2 ^a^	45.8 ^b^	48.9 ^b^	1.52	0.001	73.0 ^a^	55.3 ^b^	59.4 ^b^	1.39	0.002	1.08	0.002	<0.001	0.037

DM, dry matter; OM, organic matter; NDF, neutral detergent fiber; ADF, acid detergent fiber; ADL, lignin; CP, crude protein; CT, condensed tannin; IVOMD, in vitro organic matter digestibility; ME, metabolizable energy; DMI, dry matter intake; LW, live weight; S, season; Y, year; SEM, standard error of means. For the same year, means with different lowercase letters (a–c) in the same row indicate significant differences (*p <* 0.05). ^1^ DMI was calculated considering the seasonal variation in the LW of each experimental goat.

**Table 3:** Temporal variations in grazing activity of experimental dairy goats browsing in a Mediterranean woodland in northern Morocco.

	Dry Year (2016)					Wet Year (2017)						*p*-Value		
	Spring	Summer	Autumn	SEM	*p*-Value	Spring	Summer	Autumn	SEM	*p*-Value	SEM	S	Y	S × Y
**IceTag Data**														
Lying (%)	3.9 ^c^	13.1 ^a^	10.7 ^b^	0.556	<0.001	5.3 ^c^	15.4 ^a^	9.10 ^b^	0.423	<0.001	0.312	<0.001	<0.001	<0.001
Standing ^1^ (%)	96.1 ^a^	86.9 ^b^	89.3 ^b^	0.919	0.002	94.7 ^a^	84.6 ^c^	90.9 ^a^	0.821	<0.001	0.727	<0.001	0.003	0.008
Steps (×1000)	4.92 ^b^	7.15 ^a^	7.78 ^a^	0.325	0.001	5.15 ^c^	6.58 ^b^	7.45 ^a^	0.288	<0.001	0.216	<0.001	0.002	0.010
**GPS collar data**														
Horizontal distance (km/day)	4.36 ^b^	6.89 ^a^	7.76 ^a^	0.125	<0.001	4.62 ^b^	6.15 ^a^	7.06 ^a^	0.139	<0.001	0.119	<0.001	0.002	<0.001
Vertical distance (km/day)	0.304 ^b^	0.605 ^a^	0.588 ^a^	0.054	<0.001	0.345 ^b^	0.554 ^a^	0.510 ^a^	0.065	<0.001	0.049	<0.001	0.001	<0.001
Speed (m/s)	0.198 ^a^	0.151 ^c^	0.170 ^b^	0.006	<0.001	0.213 ^a^	0.144 ^c^	0.195 ^b^	0.008	<0.001	0.005	<0.001	<0.001	<0.001
**CART analysis data (%)**														
Grazing	57.2 ^a^	39.2 ^b^	41.2 ^b^	0.789	0.002	59.1 ^a^	35.7 ^c^	44.9 ^b^	0.868	<0.001	0.673	<0.001	<0.001	<0.001
Resting while standing	23.2 ^a^	21.0 ^ab^	18.7 ^b^	0.940	0.018	25.4 ^b^	30.1 ^a^	22.3 ^b^	0.818	0.003	0.002	0.013	0.023	0.035
Walking without grazing	15.7 ^c^	26.7 ^b^	29.4 ^a^	0.728	<0.001	10.2 ^c^	18.8 ^b^	23.7 ^a^	0.902	<0.001	0.644	<0.001	<0.001	0.047

S, season; Y, year; SEM, standard error of means. For the same year, means with different lowercase letters (a–c) in the same row indicate significant differences (*p* < 0.05). ^1^ Standing includes grazing, resting-while-standing and walking times.

## Data Availability

The data presented in this study are available from the corresponding author on request.
